# Presurgical video-EEG monitoring with foramen ovale and epidural peg electrodes: a 25-year perspective

**DOI:** 10.1007/s00415-022-11208-6

**Published:** 2022-06-15

**Authors:** Gadi Miron, Christoph Dehnicke, Heinz-Joachim Meencke, Julia Onken, Martin Holtkamp

**Affiliations:** 1 Epilepsy-Center Berlin-Brandenburg, Institute for Diagnostics of Epilepsy, Berlin, Germany; 2 Epilepsy-Center Berlin-Brandenburg, Department of Neurology, Charité – Universitätsmedizin Berlin, Berlin, Germany; 3 Department of Neurosurgery, Charité – Universitätsmedizin Berlin, Berlin, Germany

**Keywords:** Epilepsy surgery, Foramen ovale, Epidural peg electrodes, Lateralization, Postsurgical outcome, Seizure onset zone

## Abstract

**Background:**

Epilepsy surgery cases are becoming more complex and increasingly require invasive video-EEG monitoring (VEM) with intracranial subdural or intracerebral electrodes, exposing patients to substantial risks. We assessed the utility and safety of using foramen ovale (FO) and epidural peg electrodes (FOP) as a next step diagnostic approach following scalp VEM.

**Methods:**

We analyzed clinical, electrophysiological, and imaging characteristics of 180 consecutive patients that underwent FOP VEM between 1996 and 2021. Multivariate logistic regression was used to assess predictors of clinical and electrophysiological outcomes.

**Results:**

FOP VEM allowed for immediate resection recommendation in 36 patients (20.0%) and excluded this option in 85 (47.2%). Fifty-nine (32.8%) patients required additional invasive EEG investigations; however, only eight with bilateral recordings. FOP VEM identified the ictal onset in 137 patients, compared to 96 during prior scalp VEM, *p* = .004. Predictors for determination of ictal onset were temporal lobe epilepsy (OR 2.9, *p* = .03) and lesional imaging (OR 3.1, *p* = .01). Predictors for surgery recommendation were temporal lobe epilepsy (OR 6.8, *p* < .001), FO seizure onset (OR 6.1, *p* = .002), and unilateral interictal epileptic activity (OR 3.8, *p* = .02). One-year postsurgical seizure freedom (53.3% of patients) was predicted by FO ictal onset (OR 5.8, *p* = .01). Two patients experienced intracerebral bleeding without persisting neurologic sequelae.

**Conclusion:**

FOP VEM adds clinically significant electrophysiological information leading to treatment decisions in two-thirds of cases with a good benefit–risk profile. Predictors identified for electrophysiological and clinical outcome can assist in optimally selecting patients for this safe diagnostic approach.

**Supplementary Information:**

The online version contains supplementary material available at 10.1007/s00415-022-11208-6.

## Introduction

In selected patients with focal drug-resistant epilepsy (DRE), surgical removal of the epileptogenic zone is the most efficacious therapy. The aim of preoperative assessment is to delineate the tissue that needs to be removed, and to define the postsurgical benefit–risk ratio. In some cases, electrophysiological information derived from scalp video-EEG monitoring (VEM) is insufficient to reach this aim [1]. One limitation of scalp EEG is poor spatial localization of the seizure onset zone. Ictal events originating from deep or interhemispheric structures may not be visible on scalp EEG, thus providing no additional information, or may be misleading as the epileptic activity can appear bilaterally or contralaterally to the epileptogenic zone. This is particularly challenging in temporal lobe epilepsy, due to the tendency of seizures for prompt bilateral spread and due to low sensitivity of scalp EEG to electrical signals generated in mesial temporal structures [2]. Another limitation of scalp EEG is that physiological artifacts, e.g., by muscle activity or movement, may obscure ictal onset and interpretation of findings [1, 3].

Thus, non-invasive investigations at times must be supplemented by subsequent intracranial EEG (iEEG) diagnostics. The ILAE-defined indications for iEEG include to delineate the epileptogenic zone when prior non-invasive workup was inconclusive and to resolve divergence of non-invasive data pointing to several possible epileptogenic sources [4]. The most common iEEG diagnostics in use include stereotactic depth EEG (SEEG) and subdural electrodes (SDE). A main advantage of SDE is that it provides wide coverage of neocortical targets and that eloquent cortical structures can be defined by electrical stimulation; furthermore, this recording approach can also assist intraoperatively [4]. SEEG can be implanted bilaterally providing electrographic information from deep brain structures. These procedures, although highly informative, pose increased risks to the patients including a major complication rate of 9.6% for SDE implantation and 4.4% for SEEG [5]. Furthermore, studies have reported a detrimental cognitive effect caused by hippocampal depth electrodes implanted in the unaffected side [6, 7]. These complication rates may contribute to the fact that up to 50% of potential surgical candidates reject their treating physicians’ recommendations for implementation of iEEG investigations, thus excluding these patients from highly effective surgical therapy [8].

A diagnostic approach using foramen ovale (FO) and epidural peg electrodes (FOP) likely reduces risks to the patients. In this approach, electrodes are inserted through the FO and thus placed in close proximity to mesial temporal structures, while peg electrodes are inserted epidurally via burr holes in the skull over hemispheric regions. The electrophysiological rationale for utilizing FOP diagnostics is that FO electrodes can provide good sampling of mesial temporal structures and thus lateralization of the seizure onset zone [9, 10], while peg electrodes may give lateralizing and in some cases additional rough localizing information from the convexities. A limitation of this approach is that FO electrodes cannot sample deep-seated temporal structures, whereas peg electrodes are limited to superficial cortical regions. Compared to scalp VEM recording, FOP electrode recordings have reduced interference due to physiological artifacts including muscle, blinking, and movement.

Since first reported over 30 years ago [11, 12], several studies have described the clinical utility and safety of FOP VEM [9, 11, 13–20]. These studies have reported positive results as to the clinical utility; however, they have mostly focused on FO electrodes in patients with mesial temporal lobe epilepsy. In this population, the most recent studies reported that a treatment decision for or against resection could be reached in 75–90% of cases [18, 19]. Studies examining combined FO and peg electrode recording are scarce, mainly describing experience from 1990s, a period different with regard to availability of modern day neuroimaging diagnostics including high resolution MRI [12, 21, 22]. Furthermore, the extent to which these methods can provide meaningful information in patients with extratemporal epilepsy is yet unexplored.

At the Epilepsy-Center Berlin-Brandenburg, a presurgical VEM approach utilizing FO and additional peg electrodes has been in regular use since the mid-1990s. Thus, after 25 years of experience, a unique cohort of patients has accumulated, allowing for reappraisal of the clinical utility of this method. This study aims to give a broad overview of the advantages and limitations of a presurgical FOP VEM approach.

## Methods

### Study population

We conducted a retrospective analysis of all patients that underwent VEM with FO and/or peg recordings between June 1996 and August 2021 at the Epilepsy-Center Berlin-Brandenburg. During the study period, patients for whom noninvasive scalp VEM was insufficient for determination of the seizure onset zone underwent either FOP or invasive VEM. The diagnostic modality was tailored to the patient and chosen during patient case conferences comprised of a multi-disciplinary team with epileptologists, neuropsychologist, and neurosurgeons. For each patient, FOP VEM was performed after non-invasive assessment including scalp VEM, during which the following clinical data were collected: sex, age at epilepsy onset, age at FOP VEM, total number of current and previous anti-seizure medications (ASM), seizure semiology, presumed allocation to temporal or extratemporal epilepsy, number of disabling seizures per month at time of FOP VEM, findings on MRI and PET, fMRI or WADA test results, scalp VEM ictal and interictal findings and indication for FOP VEM. Clinical outcome and data collected from FOP VEM included: side and region of ictal onset, presence and lateralization of interictal epileptiform activity (unilateral, bilateral or no activity recorded), physicians’ surgical treatment decisions, 1-year postsurgical seizure outcome, and histopathology. Patients’ postsurgical outcome was categorized based on seizure freedom. Patients who following FOP VEM were recommended additional invasive EEG underwent either SDE or SEEG (the former modality was available in our center since the beginning of the study period, the latter was first implemented in 1999). Reports of all surgery conferences prior to and following FOP VEM were reviewed to determine indication for monitoring and treatment decisions.

Seizures with clear ictal onsets were defined as those that had a definite electrophysiological onset on one side and/or in one region. Unclear ictal-onset seizures were defined as those that had simultaneous bilateral appearance or were otherwise deemed nonlateralizable by the treating epileptologists. Ratios of clear ictal onsets were calculated per patient by dividing the number of seizures with a clear ictal onset by the number of total seizures recorded. For each patient, ratios were calculated both for scalp and FOP VEM. Patients for whom over 50% of recorded seizures during VEM were of unclear ictal onset were considered to have nonlateralizable onset, otherwise patients had unilateral onset if all seizures arose from the same side, or bilateral onset if seizures arose separately from both the right and left hemispheres.

This study was approved by the Institutional Review Board of Charité – Universitätsmedizin Berlin. Due to the retrospective nature of the study and due to the fact that all procedures were part of routine care, informed consent of patients was waived.

### Magnetic resonance imaging

All patients underwent cMRI (either 1.5 or 3 T) as part of clinical routine including 3D T1, transversal T2, transversal and coronal FLAIR, and a hemo-sensitive sequence (T2* or SWI).

### FO and peg electrode placement procedure, resection procedures

The location of peg electrodes was tailored per patient and decided on during patient case conferences, and according to the treating team‘s hypothesis regarding the seizure onset zone. In general, peg electrodes covered the convexities of temporal, frontal and/or parietal lobes. One hundred and fifty three patients underwent FOP VEM with combined FO and peg electrodes, 18 patients had only peg electrodes, and 9 patients had only FO electrodes, the latter in addition to scalp electrodes. Peg-implanted patients had an average of 8.1 ± 2.4 (range 4–12) electrodes. Implantation and removal of electrodes and, if patients were eligible and consented, resective surgery were done at the Department of Neurosurgery at the Charité—Universitätsmedizin Berlin. FO implantation was done using a previously described approach [23]. Patients underwent induction of general anesthesia, and were placed supine with the head at 45°. At this point, the FO and foramen spinosum were identified on anterior–posterior X ray imaging. A puncture of the skin 2.5 cm lateral to the angle of the lip was performed, and the needle was advanced first toward the angle of the jaw, then to the medial cantus 3.5 cm anterior to the plane of the meatus acusticus internus. Under fluoroscopic imaging guidance, the needle was then advanced toward the FO, with attention given not to puncture the mucosa. After entering the FO, the stylet was removed and the electrode inserted. Each FO electrode had three contacts. Location of the electrodes was then confirmed by fluoroscopic imaging. Finally, the needle was removed and the electrode fixated to the skin of the cheek. For peg electrode implantation, a 1.5 cm skin incision was made, and afterward a diamond drill was used to make a burr hole with a diameter of 5 mm. Here, the electrode was inserted, the position secured using bone wax placed over the burr hole, and then the wound was stitched close, with the outside part of the electrode fixated.

### EEG recordings

VEM was carried out at the Institute for Diagnostics of Epilepsy as part of the Epilepsy-Center Berlin-Brandenburg. EEG analysis was performed with the in-house developed EEG software (“Programm zum Berliner System”, Version 071,128, IDE gGmbH Berlin). 

### Statistical analysis

Continuous variables are presented as mean + standard deviation, medians are also provided in the Tables. Paired T test was used to compare noninvasive and FOP VEM clear ictal-onset ratios, McNemar’s test was used to compare paired changes in interictal activity. Continuous variables were tested with Mann–Whitney *U* test due to skewed data distributions. Categorical variables were tested with chi-square test. Variables with *p* < 0.1 were included in a binary regression analysis (inclusion method: backward stepwise; iteration 20; *p* value < 0.05), done to assess prediction of clear ictal onset on FOP VEM, resection recommendation, and 1-year postsurgical seizure freedom based on clinical and electrophysiological parameters. Statistical analysis was performed with SPSS Statistics version 28.0 (IBM, NY, USA). Raincloud plot of paired *T* test analysis was done using JASP software version 0.16, implementing stats R package.

## Results

### Noninvasive work-up and indication for FOP monitoring

The demographic, clinical, imaging, and scalp VEM characteristics are summarized in Table 1. One hundred and eighty DRE patients (99 males, age 34.1 ± 12.2 years, 11 patients were minors, age at epilepsy onset 12.6 ± 10.0 years) underwent FOP VEM during the study years. Over the study years, an average of 6.0 ± 1.5% of all patients undergoing presurgical evaluation in our center underwent FOP VEM, with an overall decline from 8.8% in the first five study years (1996–2001) to 5.4% of all presurgical evaluated patients in the most recent five years, *p* = 0.03, Supplementary Table 1. Prior scalp VEM included 9.8 ± 18.4 seizures recorded per patient, with 32.6% of patients having unilateral, 22.3% bilateral and 45.1% nonlateralizable ictal onsets. On MRI, 43.9% of patients had unilateral and 18.3% had bilateral lesions, 37.8% were nonlesional. The most common pathological MRI finding was mesial temporal sclerosis, found in 41 patients (17 right, 15 left, 9 bilateral). In 82 patients, the indication for FOP VEM was to resolve divergence of noninvasive data, and in the remaining 98 patients, to obtain additional information allowing for definition of the epileptogenic zone.

**Table 1:** Patient demographics, non-invasive work-up results

Sex, male, female, N (%)	99 (55.0%), 81 (45.0%)
Age, mean ± SD, median	34.1 ± 12.2, 34.5
Age at epilepsy onset, mean ± SD, median	12.6 ± 10.00, 10.0
Number of previous and current ASMs, mean ± SD, median	6.7 ± 2.6, 6
FBTCS, *N* (monthly rate mean ± SD, median, %)	146 (2.4 ± 8.6, 0.3, 81.1%)
FIAS monthly rate, mean ± SD, median	20.8 ± 34.2, 8
*Epilepsy localization*	
Temporal, *N* (%)	107 (59.4%)
Extratemporal, *N* (%)	73 (40.6%)
*Non-invasive VEM findings*^*a*^	
Seizures per patient, mean ± SD, median	9.8 ± 18.4, 5
Unilateral ictal onset, *N* (%)	57 (31 right, 26 left, 32.6%)
Bilateral ictal onsets, *N* (%)	39 (22.3%)
Nonlateralizable ictal onset, *N* (%)	79 (45.1%)
Unilateral interictal activity (*N*, %)	65 (44 right, 21 left, 37.1%)
Bilateral interictal activity, *N* (%)	77 (44.0%)
No interictal activity, *N* (%)	33 (18.9%)
*MRI findings*	
Unilateral lesion, *N* (%)	79 (42 right, 37 left, 43.9%)
Bilateral lesions, *N* (%)	33 (18.3%)
Nonlesional, *N* (%)	68 (37.8%)
*PET findings*	
Unilateral, *N* (%)	61 (33.8%)
Bilateral, *N* (%)	9 (5.0%)
Negative, *N* (%)	33 (18.3%)
fMRI / WADA language dominance, *N* (%)	40 (22.2%) / 13 (7.2%)
*Indication for FOP VEM*	
*Resolve divergence of conflicting noninvasive findings, N (%)*	82 (45.6%)
Conflicting scalp EEG imaging findings	37
Bilateral imaging findings	34
Multiple unilateral imaging findings	7
Conflicting MRI and pet findings	3
Clinical semiology inconsistent with EEG	1
*Define ictal onset, N (%)*	98 (54.4%)
Lateralization	55
Lateralization and localization	31
Unilateral localization	12

*N* number, *SD*- standard deviation, *ASM* anti-seizure medication, *FBTCS* focal to bilateral tonic clonic seizure, *FIAS* focal impaired awareness seizure, *VEM* video encephalographic monitoring, *MRI* magnetic resonance imaging, *PET* positron emitted tomography, *fMRI* functional magnetic resonance imaging, *WADA* intra-carotid sodium amoarbital procedure, *FOP VEM* Foramen ovale and peg electrode video encephalographic monitoring

^a^Noninvasive VEM information is available for 175 patients

### Decision-making based on FOP monitoring

**Fig. 1:**
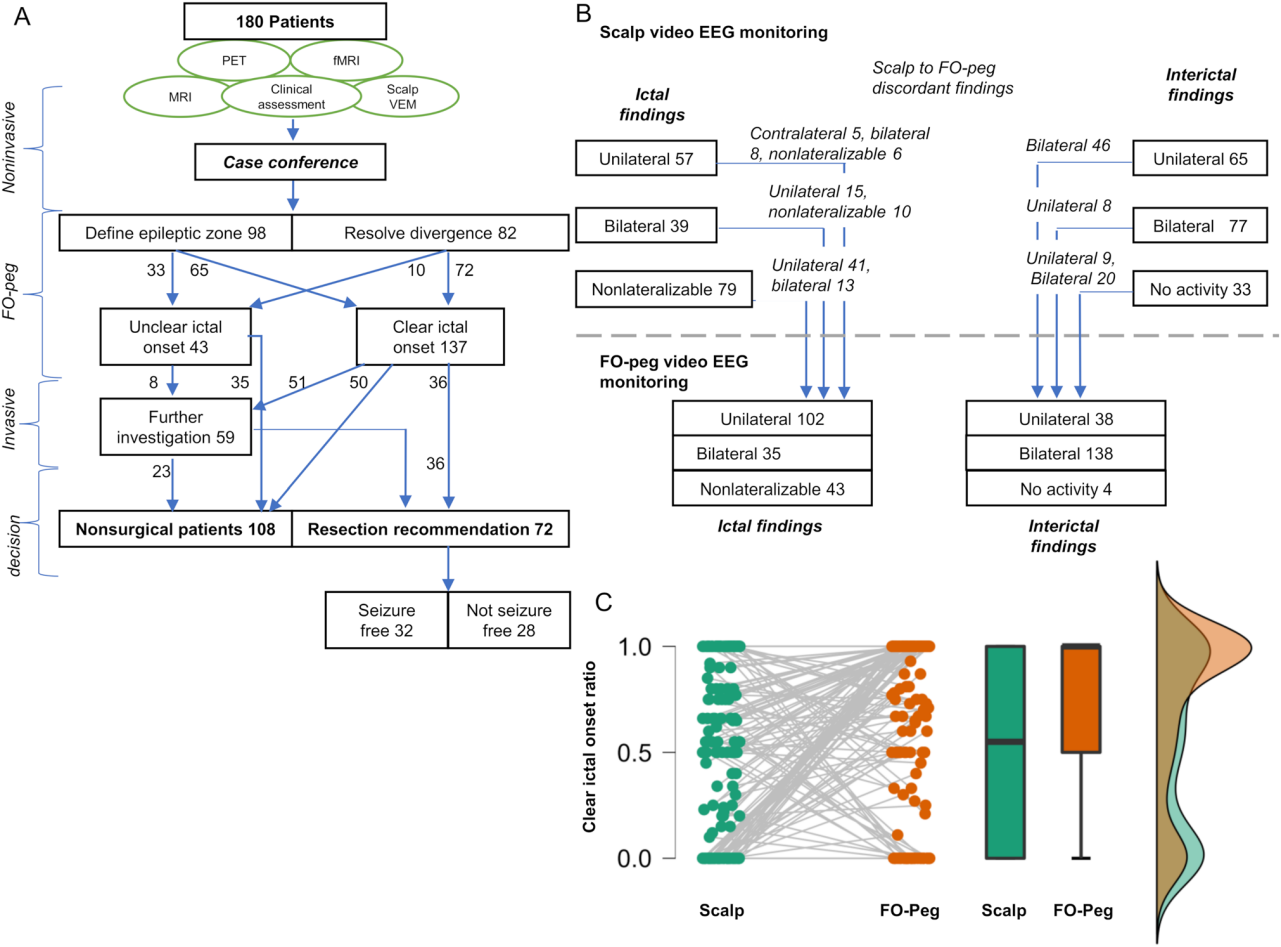
**A** Study patients flow chart. Seizure outcome follow-up 1 year after surgery was available for 60 of 68 operated patients. **B** Ictal and interictal findings during scalp (above dotted line) and FO-peg (below dotted line) video-EEG monitoring (VEM). Text above blue arrows describe discordant ictal and interictal findings on scalp and FO-peg VEM for corresponding patient groups. Scalp VEM results were available for 175 of 180 patients. **C** Paired changes in the ratio of clear ictal seizures during VEM. Each colored dot represents the ratio of seizures with a clear ictal onset (number of seizures with a clear ictal onset divided by the number of total seizures during VEM) in an individual patient during VEM, with green dots representing this ratio during scalp VEM, and orange dots during FO-peg VEM. A gray line connecting between the scalp and FO-peg VEM of each patient demonstrates the change in the ratio of clear ictal between the two investigations. Box plots and raincloud plots show distributions for scalp (green) and FO-peg (orange) VEM ratios of seizures with clear ictal onset. Box plots show in the bold black line the sample median, the hinges indicate 25th and 75th quantile, and whiskers point to 1.5 interquartile range beyond the hinges. Raincloud plot shows densities of scalp (green) and SI VEM (orange) ratios of seizures with clear onset, and the area of overlap (brown)

The findings of FOP VEM and clinical outcomes are summarized in Table 2 and Fig. 1A. FOP VEM resulted in a direct decision regarding surgical eligibility in 121 patients (67.2%), with 85 (47.2%) patients not considered surgical candidates, and 36 (20.0%) patients recommended resection. Notably, directly following FOP VEM in all patients but one, temporal lobe resections were recommended (24 right which are 66.7% of all patients recommended right-sided temporal resection, 11 left which are 42.3% of all patients recommended left-sided temporal resection). The remaining 59 patients required additional invasive work-up; however, only eight patients needed bilateral invasive investigation. In the other 51 patients, sufficient information for lateralization of the epileptogenic zone had been collected by prior FOP VEM, and thus in these patients, invasive investigation was aimed at delineating eloquent cortex or ipsilateral localization. However, 14 patients (23.7%) did not consent to additional invasive diagnostics and 3 (5.1%) were lost to follow-up after FOP VEM. Notably, a significantly higher rate of patients required an additional step of invasive diagnostics following FOP investigation during the first 5 years of the study period (24/49 (48.9%) patients, 1996–2001) compared to the following years (6/36 (16.6%) during 2002–2006, 9/33 (27.2%) during 2007–2011, 11/33 (16.9%) 2012–2016, and 9/29 (31.0%) patients during the years 2017–2021), p = 0.03.

**Table 2:** Foramen ovale and epidural peg VEM results

Number of VEM days, total (mean ± SD, median per patient)	1,803 (10.1 ± 4.7, 8)
*Ictal findings*	
Number of ictal events, total (mean ± SD, median per patient)	2,114 (11.8 ± 20.1, 7)
Clear ictal onset / unclear ictal onset^a^, N (%)	137 (76.1%) / 43 (23.9%)
Ictal onset electrode: FO/peg/both/none	41 (22.8%) / 45 (25.0%) / 66 (36.7%) / 28 (15.5%)
Number of peg electrodes per patient, mean ± SD, median	8.1 ± 2.4, 8
*Interictal findings*	
Bilateral, *N* (%)	138 (76.7%)
Unilateral, *N* (%)	38 (21.1%)
None, *N* (%)	4 (2.2%)
*FOP VEM conclusion*	
Unilateral temporal lobe seizure onset, *N* (%)	70 (37 right, 33 left, 38.9%)
Unilateral extratemporal lobe seizure onset, *N* (%)	30 (14 right, 16 left, 16.7%)
Independent bilateral seizure onsets, *N* (%)	37 (20.6%)
*Clinical decision directly resulting from FOP monitoring*	
Recommendation for resection, *N* (%)	36 (24 right temporal, 11 left temporal, 1 extratemporal, 20.0%)
Not surgical candidate, *N* (%)	85 (47.2%)
Recommendation for further invasive investigation, *N* (%)	59 (51 unilateral, 8 bilateral, 32.8%)
Recommendation for resection, end of work-up^b^, *N* (%)	72 (36 right temporal, 26 left temporal, 10 extratemporal, 40.0%)
*Postsurgical outcome, at 1 year (for N* = *60 patients available)*	
Seizure-free, *N* (%)	32 (53.3%)
Not seizure-free, *N* (%)	28 (46.7%)
*Pathology (for N* = *58 patients available)*	
MTS	25 (43.1%)
Non-MTS	33 (56.9%)
*Major complications (for N* = *136 patients available)*	
Intracranial Intracerebral bleeding, *N* (%)	2 (1.5%)
*Minor complications, N (%)*	42 (30.9%)
Unilateral trigeminal hypoesthesia	13
Self-explantation of FO electrodes	11
Trigeminal pain	8
Non-CNS infections requiring antibiotic treatment	5
Cranial nerve palsy^c^, transient	2
Swelling of skin around peg electrodes	2
Transient asymptomatic hyponatremia	1

*FOP* foramen ovale and epidural peg electrodes, *VEM* video encephalographic monitoring, *SD* standard deviation, *N* number, *FO* foramen ovale electrode, *MTS *mesial temporal sclerosis

^a^Patients were considered to have a clear ictal onset if over 50% of seizures recorded during VEM were of an identifiable brain side or region

^b^Four patients did not consent, eight patients lost to follow-up

^c^One patient unilateral hypoglossal palsy, one patient masseter weakness

After finalizing all diagnostic procedures including invasive EEG recordings, resection was recommended in 72 out of 180 patients (40.0%) including 62 temporal (36 right, 26 left), seven frontal, and three posterior cases; eventually, four patients did not consent. Patients with temporal lobe epilepsy were more likely to be recommended resection compared to patients with extratemporal lobe epilepsy (62 of 107, 57.9%, compared to 10 of 73 patients, 13.7%, *p* < 0.001). Sixty out of 68 resected patients (88.2%) had postsurgical 1-year outcome, of which 32 were seizure-free (53.3%). Fifty-eight patients had histopathology available for review, of which 25 (43.1%) had mesial temporal sclerosis (MTS). Postsurgical seizure freedom did not differ between temporal lobe and extratemporal lobe resections (27 out of 51, 52.9%, compared to 5 out of 9, 55.6%, *p* = 0.9). A nonsignificant trend toward seizure freedom was seen in patients with MTS pathology compared to non-MTS cases (15 out of 22, 68.2%, compared to 13 out of 31, 41.9%, *p* = 0.06).

### Electrophysiological information gained from FOP VEM

FOP VEM allowed determination of a clear electrophysiological ictal onset in 137 of 180 patients (38 FO onset, 44 patients peg onset, and 55 both, 76.1%), compared to 96 of 175 patients (54.9%, in five patients, VEM data were not accessible for review) on previous non-invasive VEM, *p* = 0.004. Furthermore, there was a significant increase of 34.0% per patient in the ratio of ictal events with a clear onset during FOP VEM compared to non-invasive VEM (mean 0.71 ± 0.39 vs 0.53 ± 0.42, *p* < 0.001, Fig. 1B, C), demonstrating improved detectability of ictal onset. Interictally, epileptic activity was detected on FOP VEM in 176 of 180 patients (97.7%) compared to 142 of 175 patients (81.1%) on prior non-invasive VEM, *p* < 0.001. There was a discordance found between scalp and FOP VEM ictal findings in 98 patients (56.0%), and in interictal findings in 83 (47.4%) patients, Fig. 1C.

### Factors associated with FOP VEM ictal-onset determination, recommendation for resection, and postsurgical outcomes

Univariate analysis of clinical, imaging, scalp and FOP VEM findings associated with outcomes of interest is presented in Table 3. Variables with *p* < 0.1 on univariate analysis were included in a multivariate logistic regression analysis, Table 3.

**Table 3:** Univariate and multivariate logistic regression analyses

	Clear ictal onset	Unclear ictal onset	Univariate analysis		Multivariate logistic regression analysis	
			OR	*P*- value	OR	*P*- value
*Epilepsy localization*						
Extratemporal	43	30			(reference)	
Temporal	94	13	5.0 (2.4–10.6)	< .001	2.9 (1.1–7.7)	.03
*MRI Imaging*						
Nonlesional	38	30			(reference)	
Lesional	99	13	5.2 (2.5–10.9)	< .001	3.1 (1.3–7.3)	.01
*Non-invasive VEM ictal onset*				.02		
Nonlateralizable	53	26			(reference)	.19
Unilateral	51	6			0.8 (0.1–9.1)	.84
Bilateral	30	9			0.3 (0.1v2.3)	.23
*Non-invasive VEM interictal activity*				.02		
No activity	25	8			(reference)	.25
Unilateral	57	8			1.4 (0.4–5.0)	.65
Bilateral	52	25			0.6 (0.2–1.8)	.34
Non-invasive VEM ratio of ictal events with clear onset	0.59 ± 0.40	0.33 ± 0.39		< .001	4.2 (0.3–63.9)	.30
Age at epilepsy onset	13.5 ± 10.7	9.4 ± 6.7		.03	1.0 (0.9–1.1)	.19
Number of ASMs	6.3 ± 2.3	7.6 ± 3.2		.02	0.9 (0.8–1.0)	.18
*Indication for VEM*^*a*^						NA
Defining epileptogenic zone	65	33				
Resolving divergent non-invasive investigations	72	10	3.7 (1.7–8.0)	< .001		
	**Resection recommended**	**Resection not recommended**				
*Epilepsy localization*						
Extratemporal	10	63			(reference)	
Temporal	62	45	8.7 (4.0–18.7)	< .001	6.8 (2.5–18.3)	< .001
*Imaging*						
Nonlesional	12	56			(reference)	
Lesional	60	52	5.3 (2.6–11.1)	< .001	2.1 (0.9–5.8)	.10
*FOP VEM ictal onset electrode*				< .001		
Both	17	49			(reference)	.02
FO	34	7			6.1 (2.0–18.9)	.002
Peg	21	24			2.5 (1.0–6.7)	.06
None	0	28			0	.99
*FOP VEM interictal activity*						
Bilateral	26	92			(reference)	
Unilateral	46	12	4.3 (2.0 – 9.4)	< .001	3.8 (1.3 – 11.3)	.02
FOP VEM ratio of ictal events with clear onset	0.91 ± 0.19	0.57 ± 0.44		< .001	7.4 (0.9–57.4)	.06
FBTCS monthly seizure rate^b^	0.90 ± 1.53	3.36 ± 10.93		.02		NA
*Indication for VEM*^*a*^						NA
Defining epileptogenic zone	31	67				
Resolving divergent non-invasive investigations	41	41	2.2 (1.2–4.0)	.01		
	**Postsurgical seizure-free**	**Postsurgical not seizure-free**				
*Imaging*				.06	2.4 (0.5–11.6)	.30
Nonlesional	3	8			(reference)	
Lesional	29	20				
*Additional invasive investigation*				.06	0.8 (0.2–4.1)	.83
Yes	14	19			(reference)	
No	18	9				
*FOP VEM onset electrode*				.01		
Peg	5	13			*(reference)*	.04
FO	19	7			5.8 (1.5–23.4)	.01
Both	8	8			2.5 (0.6–11.3)	.25

*OR* odds ratio, *MRI* magnetic resonance imaging, *VEM* video-EEG monitoring, *ASM* anti-seizure medications, *FOP* foramen ovale and epidural peg electrodes, *FO* foramen ovale electrode, *FBTCS* focal to bilateral tonic clonic seizures ^a^Indication for FOP VEM was not included in the multivariate analysis, as this variable is codependent on MRI imaging and scalp VEM findings

^b^Variable was not included in the multivariate analysis as 34 patients did not have FBTCS

#### Predictors of clear ictal-onset determination

Temporal lobe epilepsy (OR 2.9, *p* = 0.03) and lesional imaging (OR 3.1, *p* = 0.01) were significant for predicting clear ictal-onset determination on FOP VEM, with a model accuracy of 85.1% (*p* < 0.001, Nagelkerke R^2^ 0.35).

#### Predictors of resection recommendation

Temporal lobe epilepsy (OR 6.8, *p* < 0.001), FO electrode seizure onset (OR 6.1, *p* = 0.002), and unilateral interictal epileptic activity on FOP VEM (OR 3.8, *p* = 0.02) were significant for predicting resection recommendation, with a model accuracy of 82.7% (*p* < 0.001, Nagelkerke R^2^ 0.58).

#### Predictors of postsurgical seizure freedom

FO electrode onset was significant for predicting 1-year postsurgical seizure freedom (OR 5.8, *p* = 0.01), with an overall model accuracy of 71.7% (*p* = 0.03, Nagelkerke R^2^ 0.22). Notably, FO electrode onset was not significantly associated with MTS pathology (*p* = 0.29).

### Complications of SI monitoring

In 136 patient files, we found a direct reference to the possible presence of complications, Table 2. Two patients experienced intracerebral bleeding (1.5%), one patient had a minor hemorrhage of the left cerebral peduncle following FO electrode implementation, causing a sensory disturbance in the face and mild masticatory difficulties. Another patient experienced a minor intracerebral bleeding in the lateral temporal and parietal lobe following peg electrode insertion, causing headache. Both patients were treated conservatively. Forty-two patients had minor complications (Table 2). Length of VEM showed a trend to be shorter in patients experiencing complications compared to patients with no complications (8.8 ± 3.9 days vs 10.6 ± 4.9 days, *p* = 0.07). Among 42 patients that consented to and underwent additional SDE diagnostics, three patients (7.1%) had major complications, a higher rate compared to prior FOP VEM (*p* = 0.05). One patient had intracerebral bleeding causing contralateral weakness, and two additional patients had cerebral edema requiring ICU admittance and medical therapy.

## Discussion

In this study, we reviewed VEM findings of patients that underwent presurgical evaluation with FO and/or epidural peg electrodes at our center during the past 25 years. Our main findings are that in two-thirds of patients a clinical decision could be made directly following FOP VEM, including a recommendation for resection in one in five patients. Clinical decisions were facilitated by a significant increase in electrophysiological information on FOP VEM compared to previous noninvasive VEM. Furthermore, we identified predictors that are useful for correctly selecting patients for FOP diagnostics as well as providing prognostic postsurgical information. Finally, we report a low major complication rate during FOP diagnostics.

Previous studies have focused on the utility of FO recordings in the presurgical evaluation of patients with temporal lobe epilepsy [11, 13, 16, 18]. These included a study reporting that FO-based VEM resulted in treatment decisions in 90% of 42 adult patients with mesial temporal lobe epilepsy [19], and another study that found resection could be decided on in 45% of 38 children with previously suspected temporal lobe epilepsy [15]. Studies describing the utility of epidural peg electrode recording covering the convexities or a combined FO and peg electrode recording approach, however, are sparse. We have found no such studies of patients treated in the past 20 years, and studies describing experience in the 1990s are also limited [12, 14, 22, 24, 25]. In the largest series including 59 patients with combined peg and FO recordings, for 31% of patients, a resection was recommended, in 49% of patients additional invasive investigations were needed, and 15% of patients were disqualified from surgery [14]. Compared to these previous reports, our cohort is unique as it is larger, covers a longer time span, is composed of a more heterogenous group of DRE patients allowing for generalization of findings, and reports the use of combined FO and peg recordings. In our study, one major clinical benefit of FOP VEM was that resection could be recommended directly following the investigation among 20% of all patients and among 33% of those with temporal lobe epilepsy. In 47% of patients, resection was ruled out. The rate for resection recommendation was lower than that reported after invasive EEG investigations, with a recent comparative study finding that 78.6% of SDE patients and 66.5% of SEEG patients were likely to be resected [5]. However, postsurgical 1-year seizure freedom rate in our cohort was 53%, which is equivalent to that reported for surgery following invasive EEG diagnostics [5, 26]. Although FOP VEM lacks major advantages of invasive diagnostics, i.e., high spatial resolution, and specifically in the case of SEEG the ability to sample a multitude of deep-seated temporal structures, the equivalent post-surgical seizure freedom rate suggests that for a well-selected group of patients this diagnostic approach can provide data needed to resolve a clear hypothesis regarding the seizure onset zone, without the higher risks for complications associated with SDE or SEEG diagnostics.

**Fig. 2:**
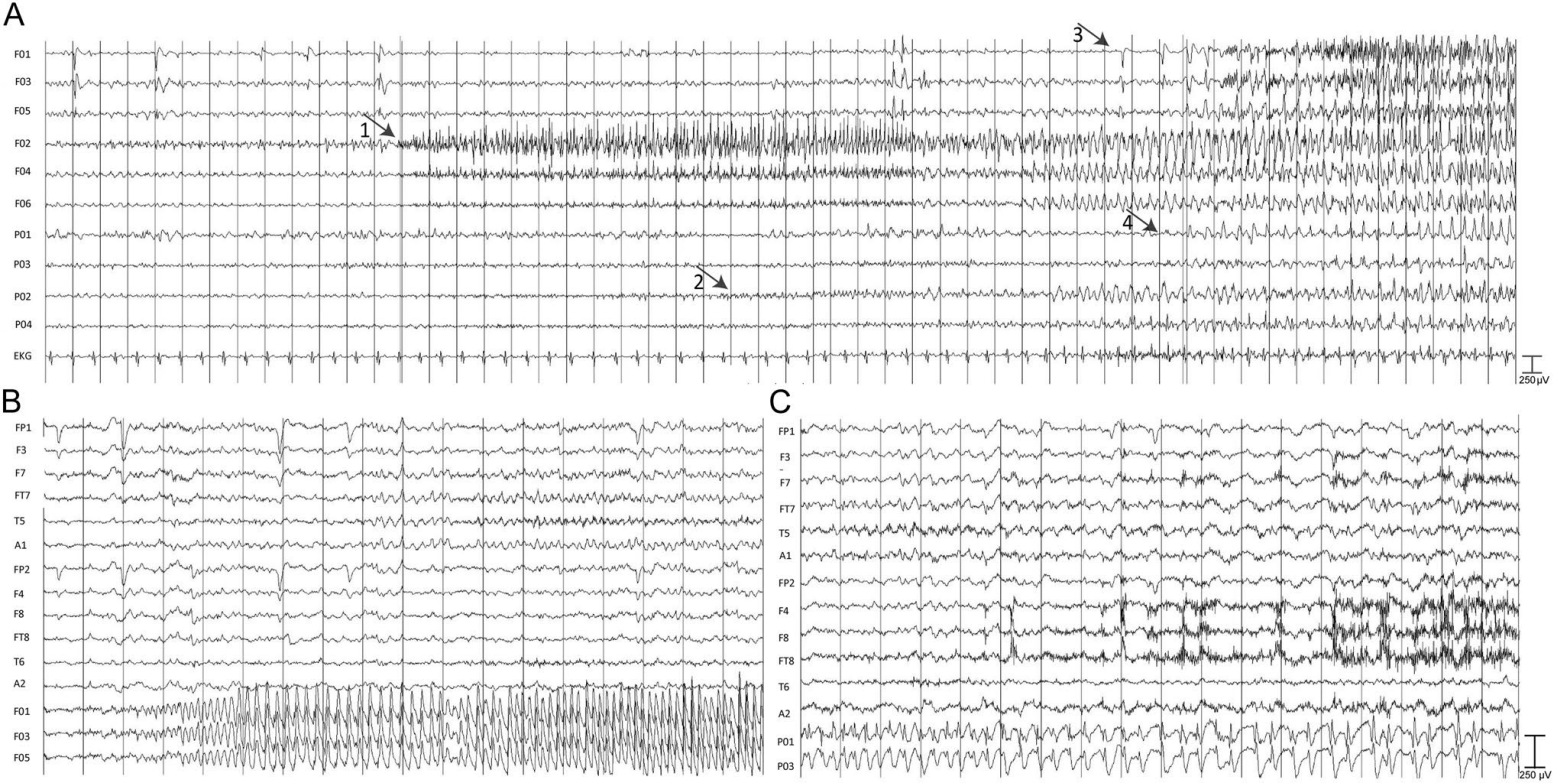
**A** In a 25-year-old female, a focal impaired awareness seizure was recorded with bilateral foramen ovale (FO; left-sided F01, F03, F05; right-sided F02, F04, F06) and epidural peg electrodes (left-sided P01, P03; right-sided P02, P04). Peg electrodes are located at F7, TP7, F8, and TP8 on a 10–10 EEG montage. Sampling rate was 2,048 Hz, time constant set at 0.2 per sec, low-pass filter 70 Hz, high-pass filter 0.3 Hz. Onset of seizure pattern is seen on right-sided FO electrodes (arrow 1), subsequently spreading to right-sided peg electrodes (arrow 2), left-sided FO electrodes (arrow 3), and left-sided peg electrodes (arrow 4). **B** In a 60-year-old male, a focal impaired awareness seizure onset was recorded with bilateral scalp electrodes covering bilateral frontal and temporal regions and left-sided FO electrodes (F01, F03, F05). Sampling rate was 2,048 Hz, time constant set at 0.3 per sec, low-pass filter 70 Hz, high-pass filter 0.3 Hz. Left-sided ictal activity with initial rhythmic theta activity rapidly evolving to rhythmic spiking is seen on FO electrodes recording from mesial temporal structures, whereas on scalp electrodes, ictal onset is not detected. **C** In a 60-year old male (same as in B), a segment of a focal impaired awareness seizure was recorded with bilateral scalp electrodes covering frontal and temporal regions and left-sided peg electrodes (P01, P03) located at FT7 and TP7, on a 10–10 EEG montage. Sampling rate was 2,048 Hz, time constant set at 0.3 per sec, low-pass filter 70 Hz, high-pass filter 0.3 Hz. Ictal activity with rhythmic spiking is seen clearly on peg electrodes, whereas on neighboring scalp electrodes, activity is less clear due to muscle artifacts and attenuated amplitude

The added electrophysiological value of FO electrodes compared to scalp for lateralization in patients with temporal lobe epilepsy has been previously described. A study examining simultaneous FO and scalp recordings of 314 seizures found that in over 40% of cases, seizures with apparent bilateral scalp onset start unilaterally on FO electrodes [9]. Another study reported that in 60% of temporal lobe epilepsy patients with bilateral or nonlateralizable scalp ictal onset, FO recordings were successful in lateralizing the epileptogenic source [17]. Our results also show significant improvement in identification of the ictal onset side and/or region on FOP VEM compared to noninvasive VEM. A clear side of ictal onset was identified in 68% of patients with previous nonlateralizable noninvasive VEM, including 30 patients (56%) that were subsequently recommended surgery. Similar to prior reports of scalp and FO VEM ictal discordance [9, 17], we found that in many cases where unilateral or independent bilateral ictal onsets are determined by noninvasive VEM, a more complex picture of the ictal onset is revealed during FOP VEM. Thus, in our study, 38% of patients with prior independent bilateral scalp ictal onsets had unilateral onset on FOP VEM, 14% of patients with prior scalp unilateral onset had bilateral ictal onset and 9% had contralateral unilateral ictal onset on FOP VEM. Although lateralization in itself is insufficient for surgical planning, as the seizure onset zone must be localized for successful resective therapy, it may provide important information for the clinician in resolving a hypothesis regarding the seizure onset zone (e.g., cases with divergent noninvasive findings). Scalp to FOP VEM discordance is possibly due to fast ictal propagation, a long latency between clinical ictal onset and electrophysiological changes on scalp EEG, or due to physiological artifacts. Interictally, we also found a significant addition of electrophysiological information, with only 2% of patients without interictal activity on FOP VEM compared to 19% on scalp VEM. Considering interictal activity was found to be a predictor of a recommendation for resection, we take this added information to be clinically valuable. Figure 2B and C demonstrates the advantage of improved sampling and reduced artifacts on FO and peg recordings in a patient with simultaneous scalp recordings.

Data derived from FO and peg electrodes served different, yet complementary roles. FO seizure onset was an independent predictor of resection recommendation and was the onset electrode in 86% of temporal lobe resections directly recommended following FOP VEM. However, in patients with clear ictal onset, peg electrodes provided additional information for excluding from surgery (4 FO and 12 peg onset, 34 both electrodes) or lateralization in patients requiring additional invasive (4 FO and 31 peg onset, 16 both) EEG investigation. In this group of patients, additional invasive diagnostics were aimed at defining eloquent cortex or further localization of the seizure onset zone. Of all 59 patients that required additional invasive testing, only in eight patients lateralization was unclear after FOP VEM, requiring additional bilateral iEEG. Thus, potentially, invasive EEG procedures in an uninvolved hemisphere aimed at epilepsy lateralization were avoided. Nevertheless, it is evident that the main advantage of FOP VEM was in decision-making of cases of temporal lobe epilepsy.

In examining trends over time of FOP VEM use, we found that in the early years of the study period, there were significantly higher rate of patients that required additional invasive diagnostics compared to the later years. One could hypothesize that this is due to advances in non-invasive investigations or experience accumulated with the use of this diagnostic approach in our center. Notably, 14 patients (23%) refused additional iEEG investigations following FOP VEM, possibly due to fatigue from procedures or due to discomfort caused by and fear toward the diagnostic process. It is possible that in these patients the opportunity for curative treatment was missed. Furthermore, one must keep in mind that those patients requiring additional invasive diagnostics following FOP VEM are exposed to more risks and incur higher medical costs, emphasizing the need for correct patient selection for this diagnostic approach.

To assess which patients are most appropriate for FOP VEM, we examined predictors of clear ictal onset and resection recommendation. Multivariate analysis revealed that temporal lobe epilepsy was an independent predictor for both these outcomes. Lesional imaging, although a predictor for identification of a clear ictal onset on FOP VEM, did not predict resection recommendation. Both ictal-onset electrode and interictal activity found on FOP VEM were, however, significant predictors of resection recommendation, emphasizing the benefit derived from additional FOP electrophysiological information for clinical decision-making.

On examination of the indications for FOP VEM, we found that an indication of resolving divergent non-invasive investigations was associated with both successful ictal onset determination and a resection recommendation. Considering that most divergent data were related to bilateral lesions on imaging or bilateral EEG ictal onsets, this suggests FOP VEM is more appropriate to answer a clear question of lateralization, rather than to find a seizure onset zone in nonlesional patients, emphasizing the need for a clear hypothesis prior to performing FOP VEM studies. However, clinicians should keep in mind the technical limitations of FOP diagnostics, specifically the fact that peg electrodes are unable to sample distant cortical areas, whereas FO electrodes are most appropriate for sampling electrophysiological signals from the mid- to posterior hippocampus, and may not be adequate for other deep-seated temporal lesions.

Providing patients with prognostic information is a central part of presurgical assessment. Previous studies have reported clinical, EEG and imaging factors associated with postsurgical outcome [27, 28], as well as a clinical algorithm for prediction of clinical prognosis [29]. We add to this literature by reporting our finding that FO electrode seizure onset was an independent predictor of seizure freedom. Notably, FO onset was not significantly associated with MTS pathology, and thus did not simply reflect the presence of this known predictor of postsurgical seizure freedom. [30] Possibly, onset on FO electrode represents early activation of mesial temporal areas of the seizure network. In any case, FO electrode onset is easily assessed during FOP VEM and could possibly be integrated in future seizure prediction models of patients undergoing FOP VEM.

We report a similar rate of major complications compared to previous studies on FO or peg electrode recordings [20], with two patients having experienced intracerebral bleeding. Notably, both patients were treated conservatively without neurologic sequalae. In contrast to previous studies reporting minor adverse events in 2.6 to 6.6 percent of patients [16, 18–20], in our cohort, there was a much higher rate of 31% (of 136 patients with information available regarding complications). This difference is possibly attributed to the fact that we had a low threshold in reporting minor adverse events including transient sensory disturbances as well as non-CNS infections indirectly related to FOP procedures occurring during the VEM. Although in 44 patient files, there was no reference to complications, we find it unlikely that these patients had complications, as in this case, there would be additional documentation or a reference on discharge letters. Thus, the rate of minor complications in our total cohort may be lower than 31% but still higher than in other reports on FOP VEM. This high rate of minor complications should be discussed with the patient prior to FOP evaluations and thoroughly considered when deciding on the most appropriate diagnostic modality. Furthermore, clinicians should take in account that complications during FOP VEM may lead to patients being hesitant to undergo additional invasive investigations, if those are needed. Compared to SDE and SEEG investigations, we report a lower major complication risk. In a recent study of SDE and SEEG in epilepsy surgery, 9.6% of patients undergoing subdural electrode recordings and 4.4% of patients undergoing SEEG experienced a major complication. An additional meta-analysis has reported that SDE carry a risk of intracranial hemorrhage in 4% of patients, as well as CNS infections in 2.3% and increased intracranial pressure in 2.4% of patients [31]. In our cohort, patients that required additional SDE testing also had a similar major complication rate of 7.1%. The use of SEEG electrodes in temporal lobe epilepsy, although associated with lower complication rates compared to SDE [32], has been reported to carry an additional risk of detrimental cognitive effects due to implantation in the unaffected mesial temporal lobe [6, 7]. Avoiding this risk is a possible advantage of FOP VEM over SEEG recordings.

An important limitation of this study is that clear ictal onset was defined according to the treating physicians, and thus is prone to observer bias. On the other hand, physicians were highly experienced epileptologists, and over the two and a half decades covered by this study, the treating physicians involved were relatively constant, diminishing interrater variability and allowing for good longitudinal assessment. Another limitation comparing non-invasive and FOP VEM findings is that most patients underwent presurgical investigations at different times, allowing for differences in pharmacological treatment regimens which could affect both interictal and ictal epileptic activity. An additional limitation is that this study is retrospective, and thus standards of clinical documentation, seizure classification definitions, and imaging quality in the beginning of the study period were different compared to recent years. Furthermore, patient files in the early years of this study were less detailed, and for 44 patients no report on presence or absence of complications was found.

## Conclusion

We report our extensive experience with presurgical assessment combining FO and epidural peg recordings in a heterogeneous population of patients with DRE. FOP VEM, in selected patients, adds clinically significant electrophysiological information leading to clear treatment decisions in two-thirds of patients, while maintaining a low complication rate. We hope our report of a favorable benefit–risk ratio of FOP VEM leads to more patients undergoing intracranial EEG investigations and subsequent resective surgery, to become seizure-free.

## Supplementary Information

Below is the link to the electronic supplementary material.

Supplementary file1 (DOCX 14 KB)
