# Supplementary material for: Presurgical video-EEG monitoring with foramen ovale and epidural peg electrodes: a 25-year perspective
Source: J Neurol. 2022 Jun 15;269(10):5474–86. doi: 10.1007/s00415-022-11208-6 (PMC9468058; doi:10.1007/s00415-022-11208-6)
Supplement: Supplementary file 1 — Supplementary file1 (DOCX 14 KB) [file 415_2022_11208_MOESM1_ESM.docx]

**Supplementary Table 1. Trends in presurgical VEM modality during study period**

|  | 1996-2001^a^ | 2002-2006 | 2007-2011 | 2012-2016 | 2017-2021^a^ |
| --- | --- | --- | --- | --- | --- |
| Total presurgical VEM, N (scalp, FOP, or invasive) | 554 | 579 | 585 | 661 | 535 |
| FOP VEM, N (% of total)^b^ | 49 (8.8%) | 36 (6.2%) | 33 (5.6%) | 33 (4.9%) | 29 (5.4%) |
| Invasive VEM, N (% of total) | 63 (11.3%) | 35 (6.0%) | 42 (7.2%) | 71 (10.7%) | 66 (12.3%) |
| Recommended Invasive VEM following FOP VEM, N (% of FOP VEM)^b^ | 24 (48.9%) | 6 (16.6%) | 9 (27.2%) | 11 (33.3%) | 9 (31.0%) |

Abbreviations: VEM - video-EEG monitoring; FOP - foramen ovale and peg electrode. Invasive VEM diagnostics include subdural and stereotactic depth EEG. ^a^ During the years 1996 and 2021, only 6 months were included in the study period. ^b^ In the years 1996-2001, a significantly higher rate of patients underwent FOP VEM (p = .03) and required additional invasive diagnostics (p = .03).
